# Involvement of lysophosphatidic acid in bone cancer pain by potentiation of TRPV1 via PKCϵ pathway in dorsal root ganglion neurons

**DOI:** 10.1186/1744-8069-6-85

**Published:** 2010-12-01

**Authors:** Hai-Li Pan, Yu-Qiu Zhang, Zhi-Qi Zhao

**Affiliations:** 1 Institute of Neurobiology, Institutes of Brain Science and State Key Laboratory of Medical Neurobiology, Fudan University, Shanghai 200032, China

## Abstract

**Background:**

It has been demonstrated that lysophosphatidic acid (LPA) released from injury tissue and transient receptor potential vanilloid 1 (TRPV1) receptor are implicated in the induction of chronic pain. In the present study we examined whether an interaction between LPA receptor LPA_1 _and TRPV1 in dorsal root ganglion (DRG) neurons contributes to the development of bone cancer pain.

**Results:**

Bone cancer was established by injection of mammary gland carcinoma cells into the rat tibia. Following the development of bone cancer pain, the TRPV1 expression and capsaicin-evoked currents were up-regulated in rat DRG neurons at L_4-6 _segments. Immunohistochemistry staining revealed a high co-localization of LPA_1 _with TRPV1 in DRG neurons. In isolated DRG neurons, whole-cell patch recording showed that capsaicin-induced currents were potentiated by LPA in a dose-dependent manner. The potentiation was blocked by either LPA_1 _antagonist, protein kinase C (PKC) inhibitor or PKCϵ inhibitor, but not by protein kinase A (PKA) inhibitor or Rho inhibitor. In the behavioral tests, both mechanical allodynia and thermal hyperalgesia in bone cancer rats were attenuated by LPA_1 _antagonist.

**Conclusion:**

LPA potentiates TRPV1 current via a PKCϵ-dependent pathway in DRG neurons of rats with bone cancer, which may be a novel peripheral mechanism underlying the induction of bone cancer pain.

## Background

Pain is the first clinical symptom of cancer in a large population of cancer patients, particularly in advanced or terminal cancer patients [1], which strongly impacts the patients' quality of life. Tumor-derived, inflammatory, and neuropathic factors may simultaneously contribute to cancer pain such as bone cancer pain [2].

Lysophosphatidic acid (LPA) is a lipid metabolite released after tissue injury, which induces diverse cellular responses including proliferation, adhesion, migration, morphogenesis, differentiation and survival [3]. Increasing evidence shows that LPA is a key mediator in cancer development including cancer cell proliferation, survival and migration [4-7]. There is a high concentration of LPA in ascitic fluid and plasma of cancer patients. Released by activated blood platelets [8], LPA promotes progression of bone metastases by inducing secretion of tumor-derived cytokine (IL-6 and IL-8) in breast and ovarian cancer cells [9,10]. Additionally, lines of study have revealed that LPA may also be a crucial factor in the initiation of neuropathic pain mediated by demyelination of peripheral nerves via activation of LPA receptor [11,12]. Six subtypes of LPA receptor, LPA_1-6_, are all G protein-coupled receptors. Three endothelial differentiation gene (EDG) family of G-protein-coupled receptors, EDG-2, EDG-4 and EDG-7 were identified as LPA receptors successively, and were named LPA_1-3 _respectively. Then p2y9 or GPR23, GPR92 and GPR87 were also identified as LPA receptors, named as LPA_4-6 _respectively [3,13,14]. Among the six subtypes, LPA_1 _receptor is the main subtype expressed in dorsal root ganglion (DRG) [11]. LPA_1 _is capable of interacting with three major G protein families, the G_i_, G_q_, and G_12 _family, resulting in the activation of their downstream cascades: mitogen-activated protein kinase (MAPK), protein kinase C (PKC) and Rho (a small GTP-binding protein)-Rho kinase, while inhibiting protein kinase A (PKA) pathway [3]. Several studies have demonstrated that LPA_1 _participates in the development of neuropathic pain through the Rho pathway [11,15,16].

In patients with advanced cancers such as breast, lung, prostate or myeloma cancer, bone pain is the most frequent cancer-induced chronic pain. However, the mechanisms underlying the development of bone pain are not completely understood. Bone cancer induces mechanical bone deformation and local tissue acidosis which may activate nociceptors via multiple molecular mechanisms, particularly by activation of the capsaicin receptor, transient receptor potential vanilloid (TRPV1). Compelling evidence has testified that TRPV1 is a critical signal molecule in the development of physiological and pathological pain [17]. It has been shown that TRPV1 is involved in the induction of bone cancer pain [18-22] and is activated by direct phosphorylation via PKC pathway [23-26], particularly via PKCε [27].

In the last decade, bone cancer pain models have been successfully established and used to explore associated mechanisms [28-30]. Given expression of LPA_1 _and TRPV1 in the DRG neurons, the present study focused on whether LPA_1 _is involved in bone cancer pain via cross-talking with TRPV1 and the possible signal pathways in the peripheral mechanism underlying bone cancer pain.

## Results

### Bone cancer-induced increase in expression of TRPV1 and capsaicin-induced currents in rat DRGs

**Figure 1:**
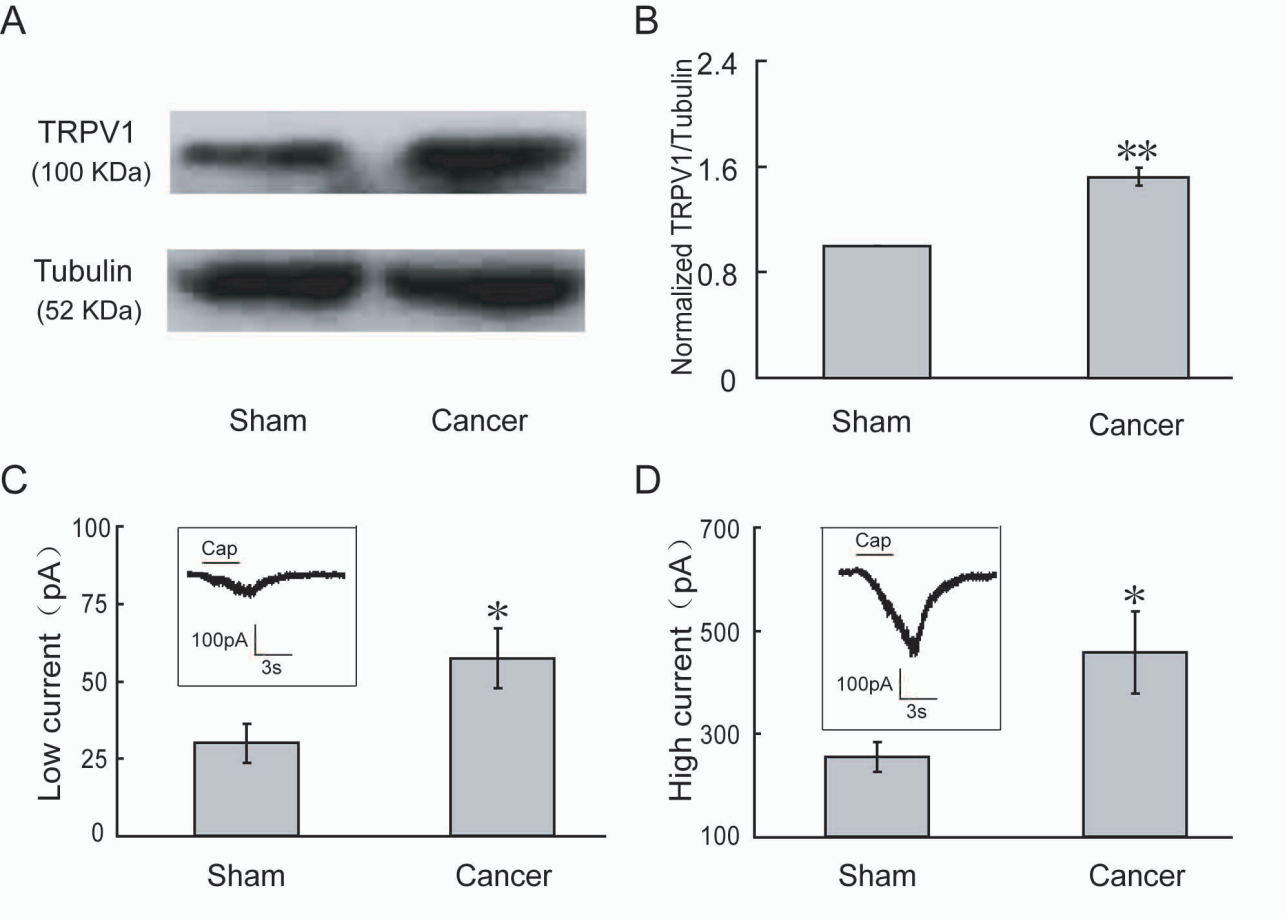
**Up-regulation of TRPV1 expression and enhancement of capsaicin-induced currents in DRG neurons of cancer rats**. Compared with sham rats, TRPV1 expression was increased in ipsilateral DRGs at L_4-6 _of bone cancer rats (**A **and **B**). **C: **In the low current amplitude group, TRPV1 currents of DRG neurons in bone cancer rats were higher than that in sham-operated rats. **D: **In the high current amplitude group, a similar result as (**C**) was obtained. Inserted figures in (**C **and **D**) represent typical currents recorded in the low and high current groups, respectively.

To elucidate the role of TRPV1 in bone cancer pain, we examined TRPV1 levels in the DRG at the L_4-6 _spinal segments 14 days after cancer cell implantation. Western blotting results showed that ipsilateral expression of TRPV1 in DRGs was higher in cancer rats (n = 6) than in sham rats (n = 6). The expression of TRPV1 was elevated by 53% ± 0.07 in the ipsilateral DRGs (DRGs from cancer rats (TRPV1/Tubulin)/from sham rats (TRPV1/Tubulin) = 1.53 ± 0.07, *p *< 0.01) (Figure 1A and 1B).

Capsaicin-evoked inward currents were recorded in L_4-6 _DRG neurons of sham-operated rats and bone cancer rats 14-16 days after inoculation. A low concentration of capsaicin was used to minimize desensitization of TRPV1. Given that repetitive administration of 500 nM capsaicin (3 s per minute four times) failed to induce detectable desensitization of TRPV1 currents [27], this concentration was used in the following tests. The percentage of DRG neurons responsive to capsaicin (0.5 μM) in cancer rats (97%, n = 33) was significantly higher than that in sham-operated rats (71.3%, n = 35). Owing to a large diversity of amplitudes of TRPV1 currents among the neurons tested, DRG neurons responsive to capsaicin (0.5 μM) were divided into the low current amplitude group and the high current amplitude group according to the amplitude of TRPV1 currents. In the former (Figure 1C), the average amplitude of TRPV1 currents in cancer rats (56.3 ± 9.41 pA, n = 14) was higher than that in sham-operated rats (29.3 ± 6.14 pA, n = 15, *p *< 0.05). Similar results were obtained in the high current amplitude group (Figure 1D). TRPV1 currents were higher in cancer rats (453.3 ± 79.48 pA, n = 14) than that in sham-operated rats (253.6 ± 28.45 pA, n = 15, *p *< 0.05). These results suggested that neurons in bone cancer rats were more sensitive to capsaicin.

### Co-localization of TRPV1 and LPA_1 _receptor in DRG neurons

**Figure 2:**
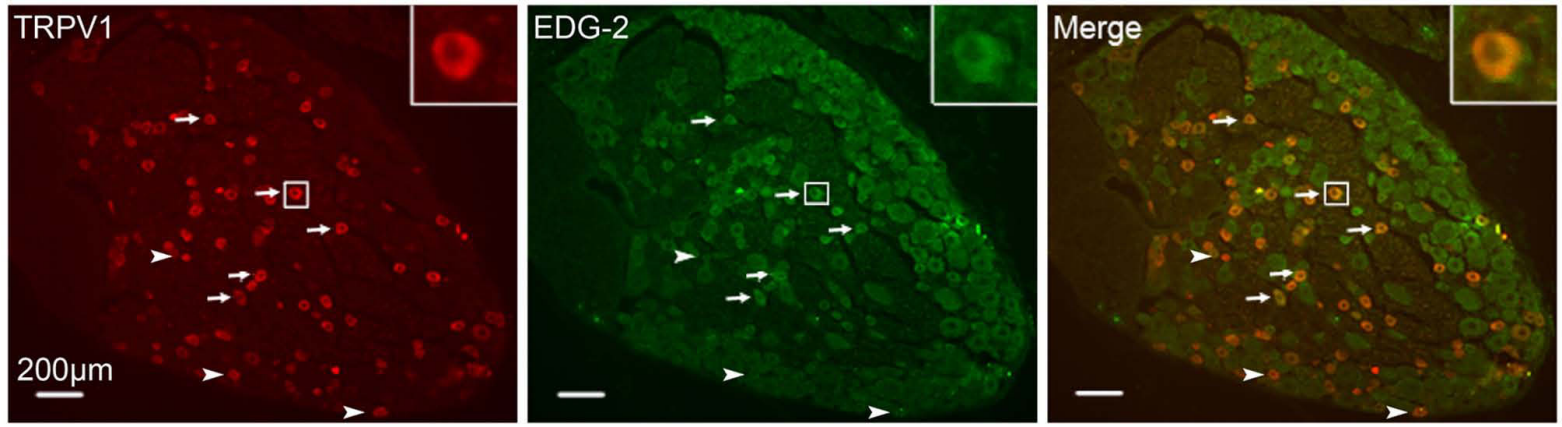
**Double immunofluorescent staining of TRPV1 (green) and EDG-2 (red) in DRG neurons**. Arrows indicate neurons positive for both TRPV1 and EDG-2. Arrowheads indicate neurons positive for TRPV1 but not EDG-2.

To investigate the possible cross-talk between TRPV1 and LPA_1_, double immunolabeling of the TRPV1 and LPA_1 _receptor (EDG-2) was detected in the DRG neurons. As shown in Figure 2 a large population of DRG neurons expressed TRPV1 (red) and EDG-2 (green). TRPV1 and EDG-2 were widely co-expressed in DRG neurons (orange). Co-localization of TRPV1 and EDG-2 in a same neuron provides the basis for their cross-talk.

### Potentiation of TRPV1 current by LPA in isolated DRG neurons

**Figure 3:**
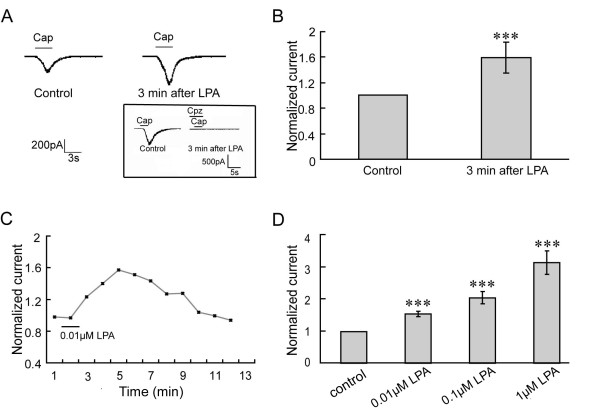
**LPA potentiates TRPV1 currents time-dependently and dose-dependently**. **A: **Representative TRPV1 current (right) potentiated by LPA in rat DRG neurons. DRG neurons were perfused with 0.01 μM LPA for 1 min before the second application of capsaicin (0.5 μM). The effect of LPA was wholly blocked by 10 μM capsazepine (cpz)**. B: **3 min after 0.01 μM LPA perfusion on DRG neurons, TRPV1 currents were highly increased (18 out of 26 cells, *p *< 0.001). **C: **After 0.01 μM LPA was perfused, TRPV1 currents were significantly increased, with maximal currents at 3 min, and decreased slowly to control level. **D: **Potentiation of TRPV1 currents by LPA at different concentrations. LPA increases TRPV1 currents dose-dependently. The currents were 3.21 ± 0.37 (1 μM, 8 out of 11 cells, *p *< 0.001), 2.09 ± 0.19 (0.1 μM, 6 out of 9 cells, *p *< 0.001), and 1.58 ± 0.09 (0.01 μM, 18 out of 26 cells, *p *< 0.001) folds higher than control currents respectively, with no effect by 0.001 μM LPA (data not shown).

TRPV1 currents were found to be potentiated by LPA perfusion (Figure 3), while totally blocked by 10 μM capsazepine (cpz), the antagonist of TRPV1, even after LPA perfusion (inserted figure in Figure 3A). LPA-induced potentiation was found in a dose-dependent manner (Figure 3D). After 1 μM, 0.1 μM or 0.01 μM LPA was perfused, TRPV1 currents were increased by 221% ± 0.37 (*p *< 0.001) in 8 out of 11 cells, 109% ± 0.19 (*p *< 0.001) in 6 out of 9 cells and 58% ± 0.09 (*p *< 0.001) in 18 out of 26 cells recorded, respectively. The currents were maximal at 3 min after LPA perfusion under all of the three concentrations. No obvious potentiation of the current by 0.001 μM LPA (n = 15) was observed (data not shown). To minimize the pharmacological effect of LPA, 0.01 μM LPA was used in the later experiments.

**Figure 4:**
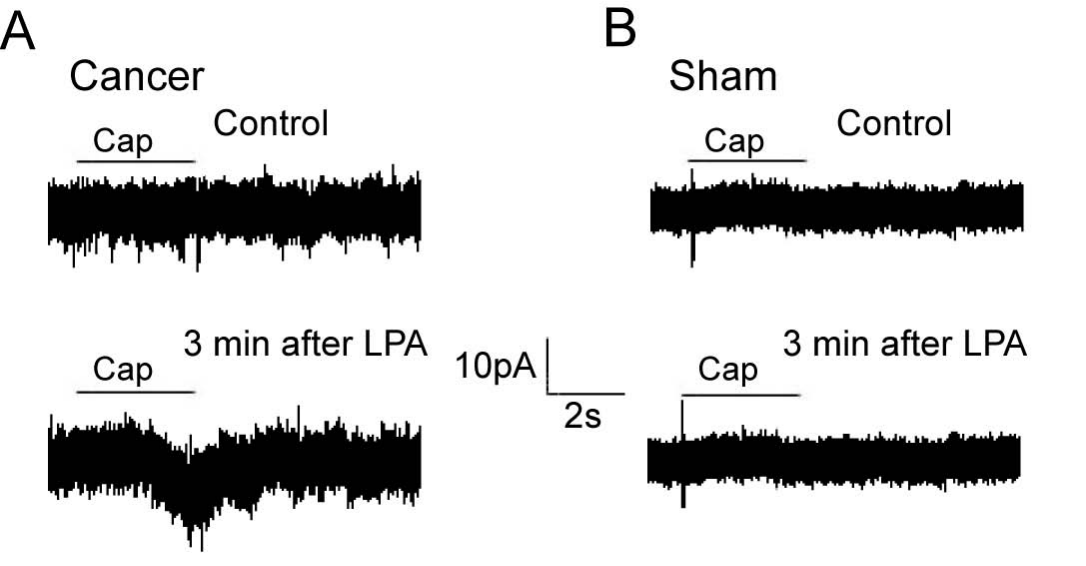
**Potentiation of TRPV1 current by LPA in DRG neurons of bone cancer rats**. **A and B: **Typical whole-cell patch recordings of DRG neurons in bone cancer and sham rats, respectively. No current was evoked by a low concentration of capsaicin (0.5 nM) in DRG neurons (the upper trace of **A **and **B**). **A: **In a bone cancer rat, capsaicin (0.5 nM) evoked a visible inward current (the lower trace) 3 min after LPA (0.01 μM) perfusion. **B: **In a sham-operated rat, no effect of LPA on application of capsaicin was observed.

Furthermore, potentiation of TRPV1 by LPA was examined in the bone cancer state. 0.01 μM LPA couldn't potentiate the TRPV1 current induced by 0.5 μM capsaicin in all of the DRG neurons (n = 24) in bone cancer rats. To avoid the possible ceiling effect, a lower concentration of capsaicin (0.05 μM) was then used. LPA (0.01 μM) could potentiate the TRPV1 current induced by 0.05 μM capsaicin in 31.58% (n = 19) DRG neurons of sham rats and 60% (n = 19) cells in cancer rats. A very low concentration of capsaicin (0.5 nM) failed to evoke TRPV1 currents in all of the DRG neurons tested (n = 11) in sham-operated rats, whereas 40% of the DRG neurons tested (n = 20) were responsive to capsaicin in bone cancer rats. We examined the effect of LPA on 12 neurons which did not respond to 0.5 nM capsaicin (sub-threshold concentration) in bone cancer rats. As shown in Figure 4A, capsaicin could evoke a TRPV1 current (the lower trace) in 8 out of 12 neurons after perfusion of LPA (0.01 μM), indicating LPA-induced potentiation of TRPV1 currents under bone cancer state. However, this phenomenon was not observed in sham-operated rats (Figure 4B, n = 11).

### PKC, particularly PKCϵ, but not PKA or Rho, mediates LPA-induced potentiation of TRPV1 currents

**Figure 5:**
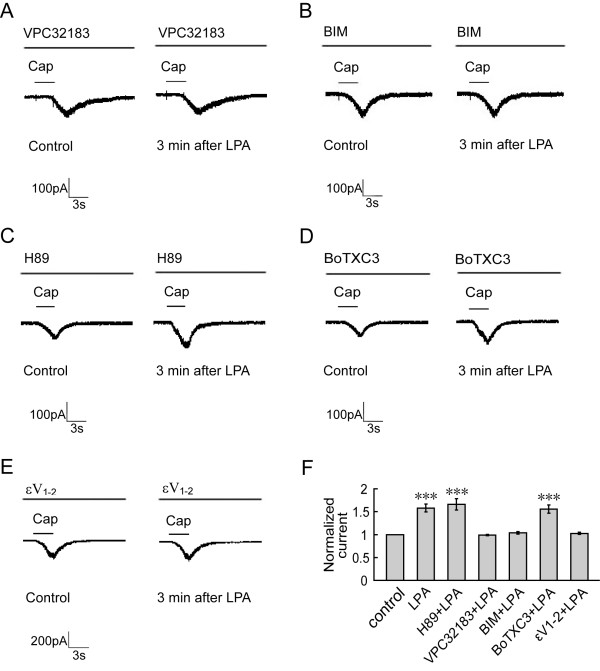
**PKC, particularly PKCε, but not PKA or Rho, involves in LPA-induced potentiation of TRPV1 currents**. Both LPA_1 _antagonist VPC32183 (1 μM, n = 15, *p *> 0.05) and PKC inhibitor BIM (1 μM, n = 13, *p *> 0.05) inhibit potentiation of TRPV1 currents induced by LPA in all of the neurons recorded (**A **and **B**). **C: **After PKA inhibitor H89 (1 μM) was delivered, no effect on LPA-induced potentiation of TRPV1 currents (n = 8, *p *< 0.001) was observed. **D: **LPA could still enhance TRPV1 currents in 14 out of 23 neurons tested (*p *< 0.001) after intracellular delivery of Rho inhibitor BoTXC3 (5 pg/μl). **E: **Intracellular delivery of PKCε inhibitor εV_1-2 _(200 μM) completely blocked LPA-induced potentiation of TRPV1 currents (n = 15, *p *> 0.05). **F: **Histogram showing summary.

Among the six LPA receptors, LPA_1_, LPA_2_, LPA_3_, LPA_4_, LPA_5 _and LPA_6_, LPA_1 _receptor is the main subtype expressed in the DRG neurons [11]. LPA failed to potentiate TRPV1 currents in all of the neurons tested after incubated with LPA_1 _antagonist VPC32183 for 30 min (n = 15) (Figure 5A, F, *p *> 0.05), suggesting that activation of LPA_1 _receptor mediates LPA-induced potentiation of TRPV1 currents.

It has been demonstrated that Rho, PKC and PKA are downstream molecules of LPA_1 _[3,31]. Their possible roles in LPA-induced potentiation of TRPV1 currents were determined. In 13 neurons tested, incubation of PKC inhibitor bisindolylmaleimide (BIM, 1 μM, 30 min) blocked potentiation of TRPV1 currents by LPA (Figure 5B, F, *p *> 0.05). However, LPA still enhanced TRPV1 currents in 8 out of 12 neurons after incubation of PKA inhibitor H89 (1 μM, 30 min, Figure 5C, F, *p *< 0.001).

Among the different PKC isoforms, PKCε is co-expressed with TRPV1 in DRG neurons and phosphorylated after inflammation [32]. Translocation of PKCε to the cell membrane triggers TRPV1 phosphorylation and sensitization [24]. Therefore, the present study further determined the role of PKCε in LPA-induced potentiation of TRPV1 current. PKCε inhibitor εV_1-2 _(200 μM) was delivered intracellularly via a recording electrode 5 min before recording. The potentiation of TRPV1 currents by LPA was completely blocked by εV_1-2 _in all of the 15 neurons tested (Figure 5E, F, *p *> 0.05), which is consistent with our previous study that PKCε inhibitor blocked substance P-induced potentiation of TRPV1 currents [27].

It is reported that Clostridium botulinum C3 exoenzyme (BoTXC3, a Rho inhibitor by ADP-riboslation) attenuated neuropathic pain at the dose of 10 pg/2 μl [33]. After BoTXC3 (5 pg/μl) was delivered intracellularly, LPA still increased TRPV1 currents in 14 out of 23 neurons (Figure 5D, F, *p *< 0.001), suggesting that Rho may not be an important signal molecule in modulation of TRPV1 by LPA. Similar results were obtained with several different concentrations, from 5 pg/μl to 5 ng/μl (data not shown).

### LPA-induced pain behaviors in bone cancer rats

**Figure 6:**
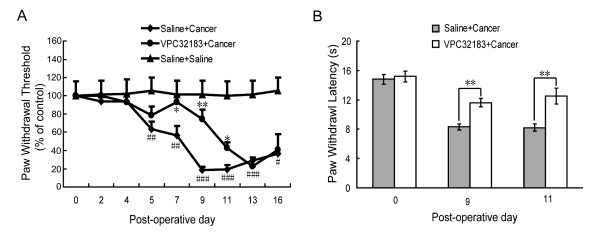
**LPA_1 _receptor antagonist VPC32183 attenuates mechanical allodynia and thermal hyperalgesia in bone cancer rats**. Injections of VPC32183 (i.t., 0.6 mM, 30 μl) or saline (30 μl) were made before cancer cell injection (D_0_), and D_2_, D_4_, D_7_, D_9 _after cancer cells injection. **A: **Alleviation of bone cancer-induced mechanical allodynia by LPA_1 _antagonist. Ipsilateral PWTs to stimuli of von Frey filaments were standardized with baseline. PWTs of Saline + Cancer rats decreased gradually 5 days after operation, significantly lower than that of Saline + Saline rats at D_5 _(*p *< 0.01), D_7 _(*p *< 0.01), D_9 _(*p *< 0.001), D_11 _(*p *< 0.001), D_13 _(*p *< 0.001) and D_16 _(*p *< 0.05). Compared with saline injection, administration of LPA_1 _antagonist VPC32183 increased PWTs at D_7 _(*p *< 0.05), D_9 _(*p *< 0.01) and D_11 _(*p *< 0.05) after cancer cell injection. **B: **Ipsilateral PWLs to radiant heating were increased by VPC32183 at D_9 _(*p *< 0.01) and D_11 _(*p *< 0.01) after cancer cell injection. #: (Saline + Cancer) vs. (Saline + Saline); *: (VPC32183 + Cancer) vs. (Saline + Cancer).

To investigate the functional significance of activation of LPA_1 _in the bone cancer state, LPA_1 _antagonist VPC32183 was used to examine roles of LPA in bone cancer pain. Bone cancer rats received a lumber puncture of VPC32183 (intrathecal (i.t.), 0.6 mM, 30 μl, n = 8) or saline (30 μl, n = 8) before cancer cell injection (D_0_), and D_2_, D_4_, D_7_, D_9 _after cancer cell injection. Ipsilateral paw withdrawal threshold (PWT) to stimuli of von Frey filaments and paw withdrawal latency (PWL) to radiant heating were measured as nociceptive indexes. As shown in Figure 6A, PWTs gradually decreased after injection of cancer cells, exhibiting the development of mechanical allodynia. Administration of VPC32183 significantly attenuated mechanical allodynia for about 5 days. Similarly, VPC32183 significantly attenuated thermal hyperalgesia in bone cancer rats. At D_9 _(*p *< 0.01) and D_11 _(*p *< 0.01) post-operation, PWLs of the cancer rats that received VPC32183 injection were longer than that of the cancer rats that received saline injection (Figure 6B).

## Discussion

The transient receptor potential vanilloid subtype 1 (TPRV1) is predominantly expressed in C-fiber nociceptors and responds to capsaicin, noxious heat, protons, and various endogenous ligands [34,35]. Recent studies have revealed that TRPV1 is involved in the development of cancer-induced pain [18-22]. The present study provided further evidence that capsaicin-induced currents and expression of TRPV1 in DRG neurons were up-regulated in bone cancer rats. A line of studies have testified that capsaicin-induced TRPV1 currents in DRG nociceptive neurons are enhanced by different inflammatory mediators such as bradykinin, chemokine and substance P etc. [27,36-39]. LPA was released from diverse sources such as blood platelets, cancer cells and the surrounding tissues. This inflammatory mediator contributed to the initiation of neuropathic pain associated with demyelination in the dorsal root [11]. Our recent studies exhibited that following the development of bone cancer by injection of cancer cells into the tibia, the posterior articular nerve directly innervating the region of bone cancer was severely degraded and the activating transcription factor 3 (ATF3, a marker of nerve injury and stress) expression was up-regulated in DRG neurons at spinal L4-5 segments [40]. Moreover, there was almost no spontaneous firing in the ipsilateral posterior articular nerve of bone cancer (unpublished). Noticeably, the sural nerve innervating the adjacent region of the bone cancer was intensely sensitized [40]. Therefore, capsaicin-induced responses in this study were more likely recorded from uninjured, but not injured, DRG neurons. Considerable evidence has shown that the injured nerve fibers could increase excitability of adjacent uninjured fibers which contributes to the induction of neuropathic pain [41-43]. Given that bone cancer pain characterizes neuropathic pain, it is conceivable that LPA-induced potentiation of TRPV1 current reflects an increase in excitability of uninjured fibers innervating the adjacent region of osteocarcinoma. The facilitation of adjacent uninjured fibers may play a crucial role in the induction of bone cancer pain.

Amongst six subtypes LPA_1-6_, LPA_1 _receptors is the main subtype expressed in DRG neurons and activated under the neuropathic pain state [11,15,44]. Consistent with alleviation of neuropathic pain [11], VPC32183, a LPA_1 _and LPA_3 _receptors antagonist, blocked LPA-induced potentiation of TRPV1 currents in DRG neurons, mechanical allodynia and thermal hyperalgesia in bone cancer rats, suggesting that activation of LPA_1 _receptors mediates bone cancer-induced sensitization of DRG nociceptive neurons and pain hypersensitization. A study reported that LPA_3 _receptors were expressed in some DRG neurons and knockout of LPA_3 _could relieve pain [45]. Therefore, in addition to LPA_1_, LPA_3 _may also contribute to bone cancer pain. The finding that LPA increases intracellular calcium concentration in DRG neurons supports the idea that LPA directly activates DRG neurons via LPA receptors by cross-talking with TRPV1 [46]. The findings that co-localization of TRPV1 and LPA_1 _receptor EDG-2 and bone cancer induced an increase in LPA_1 _receptor expression in DRG [40] provide further support for these phenomena.

Compelling evidence demonstrates that PKC regulates sensitivity of nociceptors through phosphorylation of many protein substrates [35,37]. Potentiation of TRPV1 currents in DRG neurons by different inflammatory mediators are mediated, at least in part, by PKC [47-51]. It has been known that five isoforms of PKC are expressed in DRG neurons, but only PKCε isoform is translocated to the cell membrane after bradykinin stimulation [52]. Our previous studies showed co-expression of TRPV1 and PKCε and inflammation-induced up-regulation of PKCε in DRG nociceptive neurons [53], and potentiation of TRPV1 currents by activation of Neurokinin 1 (NK-1) receptor via PKCε [27]. As with other G-protein-coupled receptors (GPCR) such as NK-1, LPA_1 _receptor activation-induced potentiation of TRPV1 in DRG neurons was also blocked by PKC inhibitor or PKCε inhibitor. In addition to PKC, previous studies demonstrated that PKA inhibitor blocked the potentiation of TRPV1 currents by pro-inflammatory factors such as: NGF, prostaglandins, anandamide, 5-HT, and glutamate [39,46,54-58]. However, the present study revealed that LPA-induced potentiation of TRPV1 failed to be blocked by the PKA inhibitor. Taken together, the interaction of LPA_1 _and TRPV1 is mediated by PKC, particularly by PKCε, but not by PKA. The identical process occurs in modulation of TRPV1 by activation of NK-1 in DRG neurons [27]. Therefore, it seems that PKCε, but not PKA, phosphorylation may be a common mechanism in which activation of LPA_1 _and NK-1 potentiates TRPV1 in DRG nociceptive neurons contributing to bone cancer pain and neuropathic pain. Interestingly, LPA can increase tetrodotoxin-resistant (TTX-R) sodium current [59] and induce substance P release from nociceptor endings [60]. Our recent finding showed that activation of NK-1 receptor potentiated TTX-R sodium channel Nav1.8 currents, mediated by PKCε in DRG neurons [61]. Collectively, two most important pain-related signal molecules, TRPV1 and Nav1.8, in nociceptors can be modulated by activation of LPA_1 _mainly via PKCε phosphorylation.

LPA_1 _receptors are reported to interact with members of three major G protein families, G_i_, G_q_, and G_12 _family, to regulate the activity of intracellular messenger molecules [3,62-64]. LPA_1 _couples through G_12/13 _to Rho activation, and inhibition of Rho-associated kinase, a downstream effector of LPA_1_, abolished LPA-induced action in osteoblasts [65]. An elegant study from Inoue *et al*., (2004) reported that Rho-Rho kinase signal pathway is implicated in the induction of neuropathic pain by activation of LPA_1 _receptor in DRG neurons. However, the present study showed that inhibition of Rho failed to block LPA-induced potentiation of TRPV1 currents in whole-cell recording of DRG neurons. The different results between the behavioural test and single cell recording suggest that LPA might be associated with multiple intracellular signals to modulate different targets in DRG neurons. LPA-induced potentiation of TRPV1 currents in DRG neurons is mediated by PKCε rather than Rho pathway. In addition to TRPV1, LPA could modulate other targets in the induction of bone cancer pain, which might be mediated by Rho pathway.

## Conclusion

Released by cancer cells and blood platelets, LPA not only promotes cancer cell proliferation but also contributes to the induction of bone cancer pain by enhancing TRPV1 activity in nociceptors. Understanding how cancer cells secrete and recruit cells to release LPA is significant for treatment of cancer pain. Inhibition of the LPA_1 _receptor or synthesis of LPA may be a novel therapy for cancer pain.

## Methods

### Animals

Animals used for bone cancer model and all experiments were female Sprague-Dawley (SD) rats weighting 160-200 g. Animals for subculture were female SD rats weighting 80 g. All of them were purchased from the Fudan experimental animal center. All experiments conformed to local and international guidelines on ethical use of animals and all efforts were made to minimize the number of animals used and their sufferings.

### Cancer cell inoculation surgery

Walker 256 rat mammary gland carcinoma cells were provided by Fudan University, School of medicine, department of integrative medicine and neurobiology, and were injected into the abdominal cavity of rats weighting 80 g to obtain ascitic fluid for cancer cell culture, continuously cultured from generation to generation weekly for the establishment of a bone cancer model.

Animals were anesthetized with Chloral hydrate (intra-peritoneal (i.p.) 400 mg/kg). The left tibia was chosen as the operated side. 10^7 ^carcinoma cells in 4 μl phosphate buffered saline (PBS) or 4 μl PBS alone (shame group) were injected into the tibia cavity through the knee joint. The inject site was closed by 1 μl absorbable gelatin sponge. Each animal was injected with 100,000 units of penicillin and had a two days recovery before any experiment. The detailed procedure for cancer cell culture and inoculation has been presented in previous reports [28].

### Intrathecal Injection

Rats received intrathecal injection of drugs by lumber puncture as described previously [66]. Briefly, rats were anaesthetized with isofluorane in a transparent plastic box and then placed on a roller so that the L_4-6 _vertebrae were curved. A lumbar puncture needle was introduced into the intrathecal space. The needle had been introduced intrathecally when a short flicking of the tail was observed. Then drugs were slowly injected into the intrathecal space. The needle was immediately pulled out after the injection.

### Mechanical allodynia

After 30 min acclimation in an individual testing cage, the rat's paw withdrawal threshold (PWT) was measured as the hind paw withdrawal response to von Frey hair (Stoelting, IL, USA) stimulation. In detail, an ascending series of von Frey hairs stimuli (2.0, 4.0, 6.0, 8.0, 15.0 and 26.0 g) was applied for 3 s where positive response was defined as a withdrawal of hind paw upon the stimulus, each stimulus was repeated for 5 times with a 10 min interval, and the lowest force to induce at least 3 positive responses was defined as PWT. The tester was blinded to the condition of the animals.

### Thermal hyperalgesia

As previously described [67], thermal hyperalgesia was assessed by measuring rat's paw withdrawal latency (PWL) to a radiant heat (model 336 combination unit, IITC/life Science Instruments, Woodland Hill, CA, USA). Rats were placed individually in plastic cages on an elevated glass platform and allowed for 30 min acclimation. Each hind paw received three stimuli with a 10 min interval, and the mean of the three withdrawal latencies was defined as PWL. The heat was maintained at a constant intensity. To prevent tissue damage, the cut-off latency was set at 20 s. The tester was blinded to the condition of the animals.

### Western-blotting

Animals were anesthetized with Chloral hydrate (i.p. 400 mg/kg). L_4-6 _DRGs were collected from sham and cancer animals at the 14th day after inoculation surgery, homogenized in a lysis buffer containing protease inhibitor (Sigma, St. Louis, MO, USA). The protein concentrations of each group were measured using a BCA assay (Pierce Biotechnology Inc., Rockford, IL, USA). The extracts were separated using SDS-PAGEs (10%) with 3 mg protein in each lane and transferred to PVDF membranes. The membranes were blocked in 10% non-fat dry milk for 2 h at room temperature (RT). After having been incubated overnight with goat anti-TRPV1 primary antibody (R-18, C-terminal, 1:1,000, Santa Cruz Biotechnology Inc., CA, USA) or mouse anti-Tubulin primary antibody (1:5,000, Sigma), the membranes were incubated with horseradish peroxidase (HRP)-conjugated donkey anti-goat or goat anti-mouse secondary antibody (1:3000, Santa Cruz Biotechnology) for 2 h at RT. Secondary antibody reactive bands were visualized in ECL solution (Pierce) for 1 min and exposed onto X-films for 1-5 min.

### Immunohistochemistry

Sensory neurons in L_4-6 _DRGs of normal rats were labelled by antibodies against TRPV1 and EDG-2 (endothelial differentiation gene-2, LPA_1 _receptor). Rats were given an overdose of urethane (i.p. 1.5 g/kg) and perfused intracardially with saline, followed by perfusion of 4% paraformaldehyde in 0.1 M phosphate buffer (PB, pH 7.4). L_4-6 _DRGs were then removed, post-fixed in the same fixative for 4 h at 4°c, and immersed from 10% to 30% gradient sucrose in PB for 24-48 h at 4°c for cryoprotection. Frozen 14 μm DRG sections were cut using a cryostat microtome and thaw-mounted onto gelatin-coated slides for processing. The sections were blocked with 10% donkey serum in 0.01 M PBS (pH 7.4) with 0.3% Triton X-100 for 2 h at RT and incubated for 48 h at 4°c with goat anti-TRPV1 (1:200, Santa Cruz Biotechnology) and rabbit anti-EDG-2 (1:50, Novus Biologicals Inc., CO, USA) primary antibodies in PBS with 1% normal donkey serum and 0.3% Triton X-100. Following three 10 min rinses in 0.01 M PBS, the sections were incubated in rhodamine red-X (RRX)-conjugated donkey anti-goat IgG (1:200, Jackson Immunolab Inc., West Grove, PA, US) and fluorescein isothiocyanate (FITC)-conjugated donkey anti-rabbit IgG (1:200, Jackson Immunolab) for 90 min at 4°c, and then washed in PBS. All sections were coverslipped with a mixture of 50% glycerin in 0.01 M PBS, and then observed under a Leica fluorescence microscope, and images were captured with a CCD spot camera.

### DRG neurons preparation

Acutely dissociated DRG neurons were prepared from 180 g rats as described previously [53]. L_4-6 _DRGs were removed and incubated in Dulbecco's Modified Eagle Media (DMEM), which was added with 3 mg/ml collagenase (type IA, Sigma) and 1 mg/ml trypsin (type I, Sigma), for 25 min at 36.8°C. After enzyme treatment, ganglia were rinsed three times with standard external solution, and then single cells were dissociated by trituration using fine fire-polished Pasteur pipettes. DRG neurons were placed onto coverslips (10 mm diameter) in the 3.5 cm culture dishes and incubated in Standard external solution for recordings at RT.

### Electrophysiological recordings

Whole-cell voltage-clamp recordings were made at RT (20-22°C) with an EPC-9 amplifier (HEKA Elektronik, Lambrecht/Pfalz, Germany). All recordings were performed within 2-8 h after plating. Neurons were prepared as above. Only small-diameter (15-25 μm) DRG neurons were recorded in all of the experiments. Microelectrodes were pulled by a P97 puller (Sutter Instruments, Novato, CA, USA) and those with the resistance of 2-6 MΩ were used. Standard external solution contained (in mM): 150 NaCl, 5 KCl, 2 CaCl_2_, 1 MgCl_2_, 10 HEPES, and 10 glucose, pH 7.4. The pipette solution contained (in mM): 140 KCl, 5 NaCl, 1 MgCl_2_, 0.5 CaCl_2_, 5 EGTA, 3 Na_2_-ATP, and 10 HEPES, pH 7.2. The whole cell capacitance was cancelled and series resistance was compensated (> 80%) after gigaohm seal formation and membrane disruption, and data were sampled at 5 kHz and low-passed at 1 kHz. Data acquisition was controlled by the software Pulse and Pulsefit 8.5 (HEKA Elektronik).

### Drugs

All the drugs for patch-clamp recording and intrathecal injection were purchased from Sigma, except that the LPA_1 _inhibitor VPC32183 was from Avanti Polar Lipids and PKCϵ inhibitor ϵV_1-2 _was from Biomol (Plymouth Meeting, PA). All the drugs were stocked at -20°C at a concentration at least 1000-fold the working concentration and prepared on the day of the experiment. LPA and capsaicin were applied close to the neurons by an ALA-VM8 perfusion system (ALA Scientific Instruments, Westbury, NY, USA). Capsaicin was applied once every minute and LPA was applied for 1 min. After cells were washed by standard external solution for 10 seconds, capsaicin was applied for 3 seconds, followed by standard external solution washing for 47 seconds. The control current was averaged from three continuous recordings. In the fourth minute, standard external solution was changed by LPA. Inhibitors were applied (where appropriate) to the chamber 30 min before LPA perfusion, except that BoTXC3 and ϵV_1-2 _were delivered intracellularly via a recording electrode. Inhibitors existed during the whole recording course.

### Statistical analysis

The western bolt and electrophysiology data were analyzed using student's t test. Two Way Repeated Measures ANOVA were used to testing differences of PWT and PWL values between groups. The criterion of significance was set at *, # *p *< 0.05, **, ## *p *< 0.01, ***, ### *p *< 0.001, all results are expressed as means ± standard error of the mean (SEM).

## Abbreviations

LPA: lysophosphatidic acid; TRPV1: transient receptor potential vanilloid; DRG: dorsal root ganglion; CPZ: capsazepine; PKC: protein kinase C; PKA: protein kinase A; MAPK: mitogen-activated protein kinase; EDG-2: endothelial differentiation gene-2 (LPA_1 _receptor); BIM: bisindolylmaleimide; BOTXC3: Clostridium botulinum C3 exoenzyme; I.T.: intrathecal; PWT: paw withdrawal threshold; PWL: paw withdrawal latency; ATF3: activating transcription factor 3; NK-1: Neurokinin 1; GPCR: G-protein-coupled receptor; TTX-R: tetrodotoxin-resistant; I.P.: intra-peritoneal; PBS: phosphate buffered saline; PB: phosphate buffer; RT: room temperature; HRP: horseradish peroxidase; RRX: rhodamine red-X; FITC: fluorescein isothiocyanate; DMEM: Dulbecco's Modified Eagle Media; SEM: standard error of the mean.

## Competing interests

The authors declare that they have no competing interests.

## Authors' contributions

HLP performed all of the experiments. YQZ was partially involved in experimental design and guiding. ZQZ is the corresponding author. All authors read and approved the final manuscript.
